# Adaptive nowcasting of influenza outbreaks using *Google* searches

**DOI:** 10.1098/rsos.140095

**Published:** 2014-10-29

**Authors:** Tobias Preis, Helen Susannah Moat

**Affiliations:** Warwick Business School, University of Warwick, Scarman Road, Coventry CV4 7AL, UK

**Keywords:** data science, computational social science, complex systems

## Abstract

Seasonal influenza outbreaks and pandemics of new strains of the influenza virus affect humans around the globe. However, traditional systems for measuring the spread of flu infections deliver results with one or two weeks delay. Recent research suggests that data on queries made to the search engine *Google* can be used to address this problem, providing real-time estimates of levels of influenza-like illness in a population. Others have however argued that equally good estimates of current flu levels can be forecast using historic flu measurements. Here, we build dynamic ‘nowcasting’ models; in other words, forecasting models that estimate current levels of influenza, before the release of official data one week later. We find that when using *Google Flu Trends* data in combination with historic flu levels, the mean absolute error (MAE) of in-sample ‘nowcasts’ can be significantly reduced by 14.4%, compared with a baseline model that uses historic data on flu levels only. We further demonstrate that the MAE of out-of-sample nowcasts can also be significantly reduced by between 16.0% and 52.7%, depending on the length of the sliding training interval. We conclude that, using adaptive models, *Google Flu Trends* data can indeed be used to improve real-time influenza monitoring, even when official reports of flu infections are available with only one week's delay.

## 2. Introduction

Large technological systems have now become a central part of our everyday life. By interacting with these systems, we create gigantic datasets documenting human behaviour at immense scale. The interdisciplinary field of computational social science [1,2], which aims to precisely quantify real-world social phenomena [3,4], has been fuelled by the vast amounts of ‘big data’ on human behaviour now becoming available. Recent studies in this area have started to focus on the analysis of data describing online behaviour, stemming from services such as the search engine *Google* [5–11], the search engine *Yahoo!* [12], the online encyclopaedia *Wikipedia* [13–15], the microblogging platform *Twitter* [16] and the photo-sharing website *Flickr* [17], as well as investigating data from more traditional news sources such as the *Financial Times* [18].

Traditional measurements of key social indicators, such as unemployment or housing prices, are often released with weeks or months of delay, owing to the work involved in collecting the relevant data [6]. The same applies to measurements of flu infections. In the USA, such measurements have traditionally been reported by the Centers for Disease Control and Prevention (CDC), with a typical time lag of one to two weeks. Early work suggested that instantly available data on how frequently Internet users had searched for influenza related terms may be of use in reducing this delay [19]. Ginsberg *et al.* [20] extended this line of research and demonstrated that in the USA, the relative frequencies of influenza-like illness (ILI)-related search queries on *Google* were correlated with the percentage of physician visits in which a patient presents with influenza-like symptoms. On the basis of this observation, they built a monitoring system for ILI which delivered measurements with a delay of only one day, with data accessible via the service *Google Flu Trends*.

A number of studies have built on these findings, two of which have used data stemming from *Wikipedia* [21] and *Twitter* [22] instead of search volume. However, questions have also been raised as to whether equally good estimates of current flu levels could be obtained from forecasting models using historic ILI records alone, particularly if it was assumed that CDC measurements were only delayed by one week [23,24]. In addition, concerns about structural changes affecting how *Google* presents search results to users have been raised, leading to questions about the continued usefulness of this approach [25,26].

Here, we build forecasting models which are dynamically retrained over time. Using these models, we quantify the extent to which relevant search queries aggregated in *Google Flu Trends* could have been used to improve estimates of weekly influenza levels in the USA between 3 January 2010 and 21 September 2013, beyond the forecasts which can be made from historic ILI data.

## 3. Material and methods

We retrieved the weekly unweighted percentages of patient visits due to ILI, reported through the US Outpatient Influenza-like Illness Surveillance Network (ILINet), from http://www.cdc.gov/flu/weekly/ on 10 December 2013 [27]. Here, ILI is defined as fever with a temperature of 100^°^F or greater, accompanied by a cough or a sore throat. Note that the data recorded for a given week can be updated in subsequent weeks, if the CDC have reason to believe that an updated figure would be more accurate. Here, we focus our analysis on the latest data available on the date of retrieval.

We obtained the weekly time series of query volume for searches relating to ILI symptoms from *Google Flu Trends* (http://www.google.org/flutrends) on 18 December 2013 [27]. This time series is restricted to searches made in the USA, and has been shown by Ginsberg *et al.* [20] to be correlated with the percentage of physician visits in which a patient presents with influenza-like symptoms. The creators of *Google Flu Trends* state that their algorithm for identifying influenza related searches is constantly evaluated against figures reported by the CDC and is occasionally updated to reflect changes in human online search behaviour. Since publication of the work carried out by Ginsberg *et al*., the algorithm underwent updates in 2009 and 2013 [28]. Data analysed here are therefore an amalgamation of two different *Google Flu Trends* algorithms, with the transition occurring in August 2013.

In both the patient visit and search engine query time series, weeks start on Sundays and end on Saturdays.

## 4. Results

We construct a model that can provide estimates, or ‘nowcasts’ of the percentage of patient visits due to ILI in week *t* at the end of week *t*. A simple correlation analysis confirms that the weekly *Google Flu Trends* time series is positively correlated with the weekly ILI patient visit time series (Kendall's *τ*=0.802, *z*=16.59, *n*=194, *p*<0.001, *α*=0.05). To investigate whether this correlation is sufficient to deliver more accurate measurements of ILI patient visits in week *t* than forecasts of these measurements using historic ILI patient visit data, we first build a baseline nowcasting model using historic ILI patient visits data only. To ensure the most conservative estimate of the extra value of *Google* search query data, we assume here that ILI patient visit data is always available with a delay of one rather than two weeks.

To build our nowcasting model, we use a standard approach for creating forecasting models. Specifically, we apply standard automatic model selection procedures [29] for an autoregressive integrated moving average (ARIMA) model, for the entire time period, as described in more detail by Stock & Watson [30]. Using the automatic ARIMA model selection procedures, we select a model containing three autoregressive terms and two moving average terms, both of which incorporate information about previous flu levels. This model is described as an ARIMA(3,0,2). We compare this in-sample baseline model with an advanced model, in which we add the *Google Flu Trends* time series to the ARIMA(3,0,2) as an external regressor.

**Figure 1.**
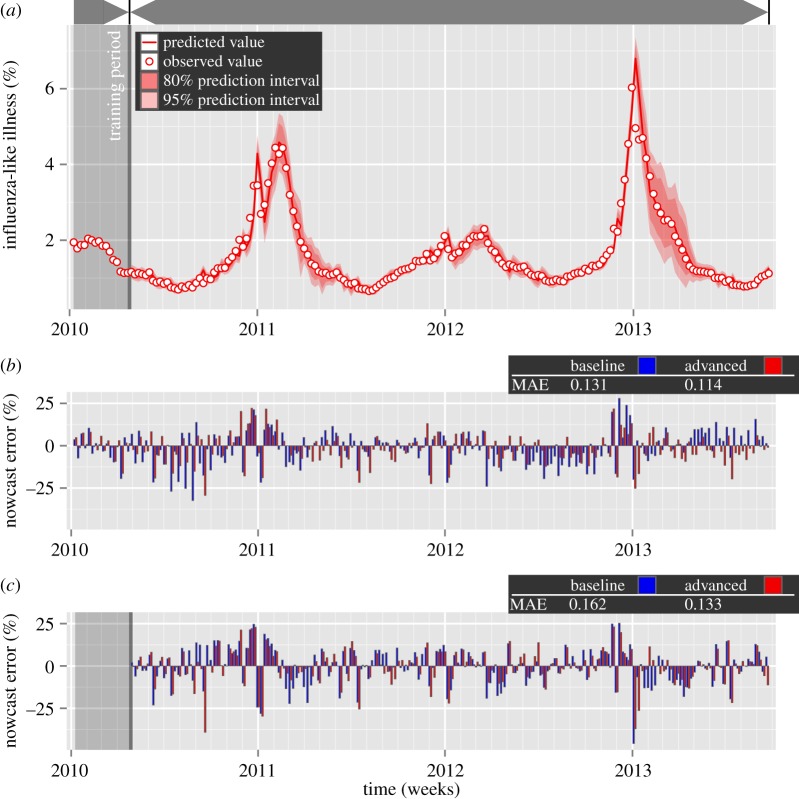
Real-time estimates (‘nowcasting’) of the unweighted percentages of weekly outpatient visits for influenza-like illness (ILI) in the USA between 3 January 2010 and 21 September 2013. Nowcasting models are forecasting models that estimate current levels of influenza, before the release of official data one week later. (*a*) Out-of-sample nowcasts using ILI data from the previous week and *Google* search query data from the current week, for a sliding training window of Δ*t*=16 weeks. (*b*) In-sample nowcast errors for the baseline model, using ILI data from the previous week only, and the advanced model, using ILI data from the previous week and *Google* search query data from the current week. (*c*) Out-of-sample nowcast errors for the baseline model and the advanced model for Δ*t*=16 weeks.

We find that the absolute residuals of the in-sample advanced model using *Google Flu Trends* data are significantly smaller than the absolute residuals of the in-sample baseline model using historic ILI patient visit data alone (median of the baseline model's absolute residuals = 0.086, median of the advanced model's absolute residuals = 0.062; *V* =11 569, *p*<0.01, *α*=0.05, two sample paired Wilcoxon-signed rank test). Figure 1*b* depicts the nowcast errors for both in-sample models. The in-sample mean absolute error (MAE) of the advanced model using *Google Flu Trends* is 14.4% smaller than the corresponding baseline model's MAE.

However, in-sample forecasting comes with its limitations. For an in-sample fit, all values from the time period between 3 January 2010 and 21 September 2013 are used to fit the model. The crucial question is therefore whether the *Google Flu Trends* time series would have significantly improved out-of-sample nowcasting, where data points are nowcast using a model trained on the previous data points. To evaluate this question, we build an out-of-sample one-step-ahead baseline model using a sliding window, where we estimate the model using data from the previous Δ*t*=16 weeks before week *t*, and then nowcast the percentage of patient visits owing to ILI in week *t*. With this approach, the optimal number of autoregressive terms and moving average terms, as well as the number of non-seasonal differences, are recalculated each week using previous data within the sliding window. We compare this out-of-sample baseline model to an advanced model which also uses the *Google Flu Trends* time series (figure 1*a*).

We find that the absolute residuals of the out-of-sample advanced model using *Google Flu Trends* data are significantly smaller than the absolute residuals of the out-of-sample baseline model using historic ILI patient visit data alone (median of the baseline model's absolute residuals = 0.095, median of the advanced model's absolute residuals = 0.075; *V* =10 728, *p*<0.001, *α*=0.05, two sample paired Wilcoxon-signed rank test).

Figure 1*c* depicts the nowcast errors for both out-of-sample models. The out-of-sample MAE of the advanced model using *Google Flu Trends* in the regression is 21.3% smaller than the corresponding baseline model's MAE, for a sliding training window length of Δ*t*=16 weeks. Qualitatively, similar results are achieved for Δ*t*=4 weeks (median of the baseline model's absolute residuals = 0.137, median of the advanced model's absolute residuals = 0.082; *V* =12 566, *p*<0.001, *α*=0.05, two sample paired Wilcoxon-signed rank test, Bonferroni correction applied), Δ*t*=8 weeks (median of the baseline model's absolute *residuals*=0.094, median of the advanced model's absolute residuals = 0.069; *V* =11 218, *p*<0.01, *α*=0.05, two sample paired Wilcoxon-signed rank test, Bonferroni correction applied) and Δ*t*=32 weeks (median of the baseline model's absolute residuals = 0.095, median of the advanced model's absolute residuals = 0.067; *V* =8605, *p*<0.01, *α*=0.05, two sample paired Wilcoxon-signed rank test, Bonferroni correction applied). Improvements of the MAEs range from 16.0% for Δ*t*=32 weeks to 52.7% for Δ*t*=4 weeks.

## 5. Discussion

In summary, we find that data from *Google Flu Trends* describing the volume of flu-related searches in a given week can be used to significantly improve estimates of the current number of influenza infections, as quantified by the number of flu-related doctor visits. Specifically, we show that *Google* search data can help improve these estimates of current levels of influenza, or ‘nowcasts’, in comparison with estimates generated by forecasts based on previous levels of influenza alone. Comparisons of an in-sample baseline model, using historic data on flu levels only, with an in-sample advanced model, augmented with data from *Google Flu Trends*, show that the MAE of in-sample ‘nowcasts’ can be significantly reduced by 14.4%. We further investigate the behaviour of an adaptive model in which the representation of the relationship between current flu levels and both *Google Flu Trends* and previous flu levels is constantly updated, and test this model out of sample. Here, we also find that an advanced model augmented with data from *Google Flu Trends* outperforms a baseline model, such that the MAE of out-of-sample nowcasts is significantly reduced by between 16.0% and 52.7%, depending on the length Δ*t* of the training interval. We conclude that *Google Flu Trends* data, combined with historic influenza levels, can indeed be used to improve real-time influenza monitoring, even when official reports of flu infections are available with only one week's delay.

## Data accessibility

Datasets used in this study are available via the Dryad Repository (doi:10.5061/dryad.r06h2).
